# Mutational dynamics across VOCs in International travellers and Community transmission underscores importance of Spike-ACE2 interaction^☆^

**DOI:** 10.1016/j.micres.2022.127099

**Published:** 2022-09

**Authors:** Priyanka Mehta, Varsha Ravi, Priti Devi, Ranjeet Maurya, Shaista Parveen, Pallavi Mishra, Aanchal Yadav, Aparna Swaminathan, Sheeba Saifi, Kriti Khare, Partha Chattopadhyay, Monika Yadav, Nar Singh Chauhan, Bansidhar Tarai, Sandeep Budhiraja, Uzma Shamim, Rajesh Pandey

**Affiliations:** a INtegrative GENomics of HOst-PathogEn (INGEN-HOPE) laboratory, CSIR-Institute of Genomics and Integrative Biology (CSIR-IGIB), Mall Road, Delhi 110007, India; b Academy of Scientific and Innovative Research (AcSIR), Ghaziabad 201002, India; c Department of Biochemistry, Maharshi Dayanand University, Rohtak, Haryana 124001, India; d Max Super Speciality Hospital (A Unit of Devki Devi Foundation), Max Healthcare, Delhi 110017, India

**Keywords:** SARS-CoV-2, Variants of concern, Genomic surveillance, Mutation analysis, Structural analysis

## Abstract

**Background:**

Emergence of SARS-CoV-2 VOCs at different time points through COVID-19 pandemic raised concern for increased transmissibility, infectivity and vaccination breakthroughs.

**Methods:**

1567 international travellers plus community transmission COVID-19 cases were analysed for mutational profile of VOCS, that led to notable waves in India, namely Alpha, Delta, and Omicron. Spike mutations in Linkage Disequilibrium were investigated for potential impact on structural and functional changes of Spike-ACE2.

**Results:**

ORF1ab and spike harboured diverse mutational signatures for each lineage. B.1.617.2 and AY. * demonstrated comparable profile, yet non-clade defining mutations were majorly unique between international *vs* community samples. Contrarily, Omicron lineages showed substantial overlap in non-clade defining mutations, signifying early phase of transmission and evolution within Indian community. Mutations in LD for Alpha [N501Y, A570D, D1118H, S982A], Delta [P681R, L452R, EFR:156–158 G, D950N, G142D] and Omicron [P681H, D796Y, N764K, N969K, N501Y, S375F] resulted in decreased binding affinity of Spike-ACE2 for Alpha and BA.1 whereas Delta, Omicron and BA.2 demonstrated strong binding.

**Conclusion:**

Genomic surveillance tracked spread of VOCs in international travellers’ *vs* community transmission. Behavioural transmission patterns of variants, based on selective advantage incurred by spike mutations, led us to predict sudden takeover of Delta over Alpha and BA.2 over BA.1 in India.

## Highlights

• The mutational landscape of 1567 international travellers and community transmission were characterized across VOCs in India
• Mutations in LD for VOCs demonstrated differentially altered binding affinity and electrostatic interactions of Spike-ACE2.
• Altered Spike-ACE2 affinity among VOCs predicted sudden takeover of Delta over Alpha and BA.2 over BA.1 in India.

## Graphical Abstract

**Figure:**
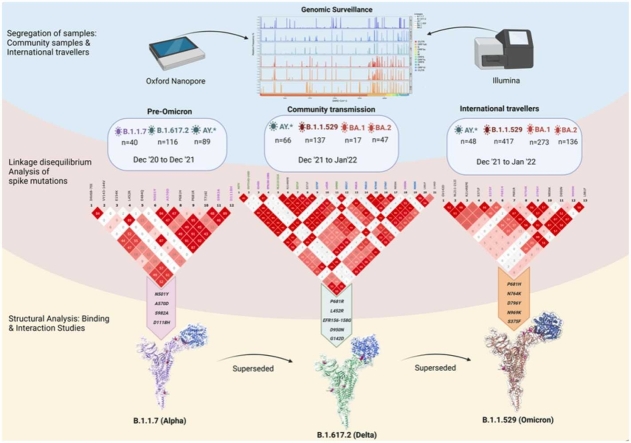

## 1 Introduction

The coronavirus disease 2019 (COVID-19) has resulted in forced implementation of unprecedented local and global travel restrictions and social distancing measures throughout the world. Despite such precautions, more than 486 million cases of COVID-19 have been reported worldwide as of March ‘22 of which ~9 million have been sequenced and shared on global data repository, GISAID ( https://covid19.who.int/, https://www.gisaid.org). As was evident during the 2014 outbreak of Ebola, which swept through the West African nations, genomic surveillance data proved extremely useful in characterising the patterns of viral transmission, monitoring the rapid accumulation of interhost and intrahost genetic variations, and, most importantly, establishing the genomic origin of the virus (Gire et al., 2014). During the Ebola outbreak, genetic data assisted in making informed response strategies and public health decisions (Kinganda-Lusamaki et al., 2021). Similarly, despite its unassuming size, worldwide genomic surveillance has aided in elucidating the epidemiological trend and mutational diversity of SARS-CoV-2 since its first reported case (Jha et al., 2021 Dec 17, Dhar et al., 2021 Jun 3, Haddad et al., 2021 May 25).

Several SARS-CoV-2 variants have been identified with Alpha variant having emerged in early September 2020 in the United Kingdom and later identified as a Variant of Concern (VOC), due to rapid transmission and enhanced virulence, leading to a negative shift in COVID-19 epidemiology. Subsequently, many different VOCs emerged from different parts of the world - Beta (South Africa), Gamma (Japan) and Delta (India). Many VOCs caused a fresh round of infections and mortality, putting further strain on public healthcare infrastructure and medical support (Kanakan et al., 2022). India also experienced waves of enhanced COVID-19 infections, the first wave between March 2020-Sept 2020 and the second one from March 2021-June 2021 (Kar et al., 2021 Jun). Alpha and Delta were the two primary VOCs observed during these waves, respectively. Recently, the Omicron (B.1.1.529) variant emerged and superseded the Delta, resulting in a new wave in India. This novel SARS-CoV-2 variant, first reported on 24th Nov 2021 in South Africa, is notable for having more than 30 mutations in the spike protein compared to a handful of mutations in the Delta and has already subdivided into two lineages, BA.1 and BA.2, since its emergence. However, despite carrying a large number of immune escape mutations, this variant quickly faded off in India with relatively milder symptoms (Dhawan et al., 2022 Mar).

The mutational landscape of SARS-CoV-2 variants that generated notable waves in India, namely the Alpha, Delta, and Omicron variants, were investigated in this work. We analysed 1567 in-house SARS-CoV-2 sequences from patients inclusive of community transmission and international travellers. We analysed the differences between the mutational profiles of community transmissions and international travellers' during the Omicron wave, identified core set of spike mutations in different VOCs and evaluated their potential structural significance. Towards this, a computational approach was used to validate the association of mutations, by performing docking and interaction studies with the ACE2 receptor.

## 2 Methods

### 2.1 SARS-CoV-2 whole-genome sequencing

Genome sequencing of the 1567 samples was done using a combination of Nanopore and Illumina sequencing platforms. Of these, 388 samples were sequenced using Oxford Nanopore Technology (ONT) library preparation protocol- PCR tiling of SARS-CoV-2 virus with Rapid barcoding (Version: PCTR_9125_v110_revB_24Mar2021). In brief, 50 ng of total RNA was taken to synthesise single stranded cDNA using LunaScript RT SuperMix (New England Biolabs, Cat. No. E3010L). The cDNA-RNA hybrid was used to amplify SARS-CoV-2 genome with rapid barcoding primers (IDT Product number: 10007184) and Q5 ® High-Fidelity 2X master mix (New England Biolabs, Cat. No. M0494S). For sequencing library preparation, the amplified products were ligated with rapid barcode sequences (SQK-RBK110.96) followed by SPRI bead purification. The purified library was then ligated with adapter protein and loaded on the MinION Mk1B/Mk1C platform.

Sequencing library preparation was also performed using Illumina COVIDSeq for 1179 samples (Cat. No. 20043675 and reference guide: 1000000126053 v04). The extracted RNA was utilised to synthesise cDNA and the viral genome was further amplified using two separate PCR reactions. The pooled amplicons then underwent tagmentation to fragment and tag amplicons with adapter sequences. This was followed by post-tagmentation cleanup and a second round of PCR amplification, ligating index adapters. The indexed amplicons were pooled and cleaned using purification beads. The pooled library was then quantified using Qubit dsDNA HS Assay kit (Cat. No. Q32854). A loading concentration of 11 pM was prepared by denaturing and diluting the libraries in accordance with the MiSeq System Denature and Dilute Libraries Guide (Illumina, Document no. 15039740 v10). Sequencing was performed on the MiSeq system, using the MiSeq Reagent Kit v3 (150 cycles) at 2 × 75 bps read length.

### 2.2 Sequencing data analysis

The ARTIC end-to-end pipeline (Artic Network [Internet]. [cited, 2022a) was used for the analysis of MinION raw fast5 files up to the variant calling. Raw fast5 files of the samples were base called and demultiplexed using Guppy basecaller, which uses the base calling algorithms of Oxford Nanopore Technologies (Nanopore Community [Internet],) with a Phred quality cutoff score > 7 on a GPU-Linux accelerated computing machine. Reads with a Phred quality score of less than 7 were discarded to filter the low-quality reads. The resultant demultiplexed fastq were normalised by a read length of 1200 (approximate size of amplicons) for further downstream analysis and aligned to the SARS-CoV-2 reference (MN908947.3) using the aligner Minimap2 v2.17 (Li, 2018). Nanopolish (Loman et al., 2015 Aug) was used to index raw fast5 files for variant calling from the minimap output files. To create consensus fasta, bcftools v1.8 was used over the normalised minimap2 output.

Fastqc was performed for all the raw fastq files generated from Illumina sequencing in order to check the Phred quality scores of all the sequences (Babraham Bioinformatics). A Phred quality score threshold of > 20 was used for filtering reads from all the samples. Subsequently, adapter trimming was performed using the Trim Galore tool (Babraham Bioinformatics) and alignment of the sequences was performed using the HISAT2 algorithm (Kim et al., 2019) on human data build hg38 to remove any human read contamination. BEDTools was used to generate the consensus fasta using the unaligned/filtered reads, and variant calling was performed using the high-quality reads (Quinlan and Hall, 2010). The sequencing depth and genome coverage for all the samples are available in Table S1.

### 2.3 Data collection and preprocessing

A total of 1567 SARS-CoV-2 samples were included in the study which were part of the Genomic Surveillance initiative of international travellers in India. All the sequences with ≥ 90% coverage and average sequencing depth > 100x were included in the study. Of the total 1567 samples, 396 samples were from pre-Omicron time points and the remaining 1171 samples were collected during the peak of the Omicron wave in India between Dec 2021-Jan 2022. Of the 1171 Omicron samples, 895 samples belonged to international travellers and 276 of community transmissions between Dec 2021 to Jan 2022.

### 2.4 Phylogenetic analysis

The Nextstrain augur pipeline (https://github.com/nextstrain/ncov) was used to perform the phylogenetic analysis of 1567 sequences. The Wuhan reference genome (MN908947.3) was used for performing multiple sequence alignment using Mafft (v7.475)(Katoh and Standley, 2013 Apr). The phylogenetic tree was constructed using IQtree (Nguyen et al., 2015 Jan). The tree was visualised at auspice.us web browser (https://auspice.us/).

### 2.5 Linkage disequilibrium analysis

To estimate inter-chromosomal linkage disequilibrium (LD), we divided the sequences into three groups based on time of sequencing and travel history as Pre-Omicron variants (n = 396) which includes VOC prevalent in India between Dec 2020-Oct 2021. Community transmission (n = 276) includes samples of COVID-19 patients without any travel history between Dec 2021-Jan 2022 during the Omicron wave and international travellers (n = 895) which included passengers who were detected COVID-19 positive on arrival at IGI airport, Delhi from different countries, between Dec 2021-Jan 2022. We used Haploview to perform LD and haplotype block analysis (Barrett et al., 2005). The plink files were manually created in excel to include all the mutations with > 1% frequency in each group. Variants with minor allele frequencies (MAF) ≥ 0.5% were used to estimate the LD. Mutations with a squared correlation coefficient (r2) ≥ 0.8 were taken for further downstream structural analysis.

### 2.6 Structural analysis

The spike mutations filtered from LD analysis for the three groups were further taken for docking studies, to see the binding affinities between spike glycoprotein and human ACE2 receptor. The fasta sequence of spike glycoprotein was taken from Uniprot (P0DTC2) and modelled using iTasser (Yang et al., 2015 Jan) whereas ACE2 protein structure was taken from PDB (PDB id:1R42). Mutations in spike glycoprotein are imparted using mutagenesis in pymol (Pymol). Rigid docking was done using High ambiguity‐driven protein–protein docking (HADDOCK) web server (van Zundert and Bonvin, 2014), where amino acids 30–41, 82–84 and 353–357 from ACE2 receptor and 437–508 from RBM of spike glycoprotein were taken as active residues (Jagtap et al., 2020). To get further insights into the docked complexes, dissociation constant (K_D_) was calculated using PROtein binDIng enerGY prediction (PRODIGY) (Vangone and Bonvin, 2015) and interactions analysis was explored through PDBsum of EMBL(Laskowski et al., 2018 Jan).

## 3 Results

### 3.1 Elucidating Pan-India epidemiology profile of prevalent SARS-CoV-2 variants

**Fig. 1:**
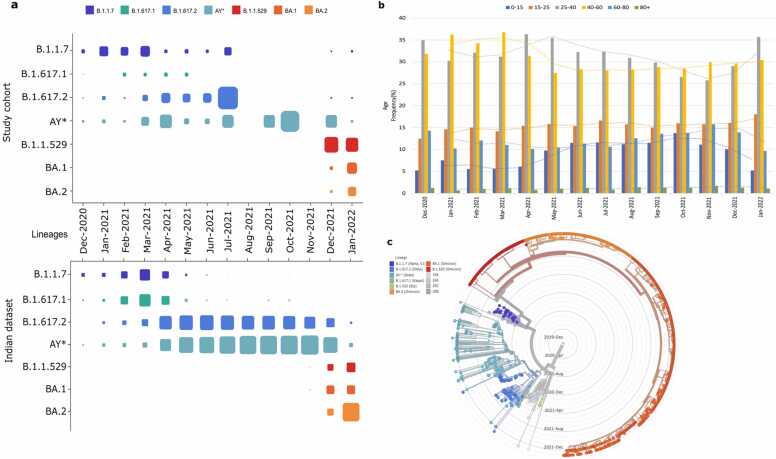
Demographics and phylogenetic evolution of the prominent SARS-CoV-2 variants in India. (a) The matrix plot demonstrates a comparative frequency distribution of SARS-CoV-2 variants between Dec 2020-Jan 2022 in the study cohort from our Lab and pan-India based on the genomic surveillance data from GISAID. (b) The age demographics of SARS-CoV-2 positive patients across India, Dec 2020-Jan 2022. (c) Phylogenetic distribution of various SARS-CoV-2 positive patients, Dec 2020-Jan 2022 in our study cohort.

SARS-CoV-2 Genomic surveillance programme helped us to collectively utilise a subset of 1567 sequences that were generated over a time from Dec 2020 to Jan 2022, to analyse the longitudinal dissemination of different SARS-CoV-2 variants. These COVID-19 positive samples include 396 individuals from Dec 2020-Nov 2021, 895 with international travel history between Dec 2021-Jan 2022 and 276 community surveillance samples between Dec 2021-Jan 2022. All the sequences included in the study have an average sequencing depth > 100x and ≥ 90% genome coverage. Genome analysis characterised 40 samples as B.1.1.7 (Alpha), 120 as B.1.617.2 (Delta), 11 for B.1.617.1 (Kappa), 555 as B.1.1.529 (Omicron), 290 for BA.1 (Omicron) and 183 for BA.2 (Omicron) lineages. Multiple different lineages for the AY (Delta sub-lineages; n = 203) were collectively grouped as AY.* . The remaining 165 samples belonged to different B.1 sub-lineages. Fig. 1a represents the epidemiological expansion of SARS-CoV-2 lineages, starting with Alpha and Kappa, followed by quick transition to and dominance of the Delta variant by March-April 2021. After July 2021, the Delta variant was superseded by the AY. * lineages, till the Omicron variant started gaining prevalence in December 2021, completely superseding the other variants in a quick span of time.

To check whether the lab specific sequencing findings capture the pan-India profile, genome analysis data submitted to GISAID over the same period from across India also showed a comparative epidemiological profile with expansion of B.1.1.7 (Alpha), B.1.617.1 (Kappa) and B.1.617.2 (Delta) lineages in 2021, and predominance of Omicron by January 2022. The difference in the frequencies of BA.1 and BA.2 amongst our study cohort and Indian dataset is attributable to substantial presence of genome sequences of Omicron from International travellers in our study cohort.

Next, we investigated the age of the Indian population affected during each of the outbreaks due to Alpha, Delta and Omicron waves. GISAID data showed age group consistency across the time scale, with 25–40 years and 40–60 years categories reflecting maximum infection. An upward trend line for the age group 25–40 years was observed during the Omicron surge, suggesting an increase in the number of younger people affected during this time, (similar observation for Delta variant), while a beginning of downward trend was evident for 60–80 age individuals. Age groups 0–15 years were found to exhibit an ascending trend between May-Nov 2021 when Delta sub-lineages were prevalent (Fig. 1b). Phylogenetic analysis shows distribution of the SARS-CoV-2 genomes across different lineages. The phylogenetic tree of our cohort samples shows two major branches. One of the branches harbours all the Delta lineages (Indian origin), whereas another branch separates the international variants, Alpha and Omicron. An increase in rate estimate from 24.867 to 29.876 subs/per/year from Alpha to Omicron wave was seen. The phylogenetic analysis proffers the evolutionary history of different lineages of SARS-CoV-2 in India. Following this, we dig deeper into the viral genome to better comprehend the genetic trend of each lineage.

### 3.2 Longitudinal mutational landscape of the prevalent strains of SARS-CoV-2

**Fig. 2:**
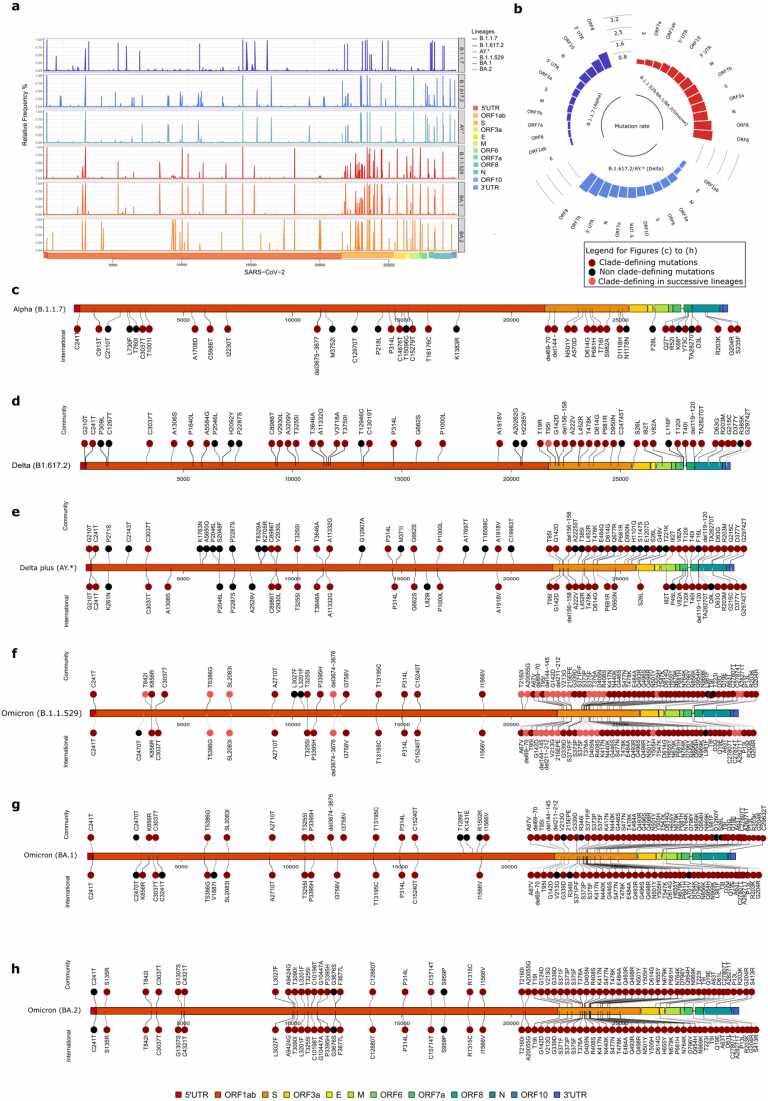
Mutational landscape of SARS-CoV-2 dominant variants across different time points. (a) A comparative pan view of the entire SARS-CoV-2 genome showing regions with high and low concentration of mutations across different VOCs. (b) Circular bar plot representing SARS-CoV-2 genes arranged in descending order of the mutation rates across Alpha, Delta and Omicron VOCs. (c) Lollipop plot of B.1.1.7 (Alpha) variant mutations from international travellers. (d) Lollipop plot of B.1.617.2 (Delta) variant mutations from community cases. (e) Lollipop plot of AY. * (Delta-sub lineages) mutations from community cases and international travellers. (f) Lollipop plot of B.1.1.529 (Omicron) variant mutations from community cases and international travellers. (g) Lollipop plot of BA.1 (Omicron) variant mutations from community cases and international travellers. (h) Lollipop plot of BA.2 (Omicron) variant mutations from community cases and international travellers. The nodes of clade-defining mutations are coloured in red for (c-h), pink nodes belong to mutations which became clade-defining in successive lineages and black nodes are for non-clade defining mutations.

We investigated the relative distribution of mutations across different SARS-CoV-2 lineages in our cohort, to obtain a comparative mutational profile and to visualise the evolution of mutations in the viral genomes. Fig. 2 highlights the similarity and uniqueness observed in the viral genomes of the lineages considered in the cohort study. The Alpha, Delta and Omicron lineages highlighted distinct mutational profiles as illustrated by Fig. 2a. Figs. 2c-2h elaborates the mutations depicted by the peaks in Fig. 2a, with the mutations present in > 10% of the samples included in the study. Starting from the 5′ UTR, C241T is a common mutation observed across all the lineages. Although ORF1ab has diverse signatures for each lineage, two mutations (C3037T/924 F and P314L) are uniformly present throughout all the lineages at high frequency. B.1.617.2 and AY. * lineages, broadly collated as Delta/plus VOC have an overall comparable profile with mutations dispersed throughout the ORF1ab locus. Similarly, B.1.1.529 and BA.1 have nearly identical mutational landscapes in ORF1ab, yet BA.2 shares very few common mutations with B.1.1.529 and BA.1 with clusters of exclusive mutations, near the 10,000 and 20,000 position on the genome. BA.2 has 36 unique mutations (29 in ORFs, 5 in spike, with 1 each in N and M protein) not found in BA.1, and lacks 33 mutations (14 in ORFs, 18 in spike with 1 in M) present in BA.1. Notably, these mutations appear to be present in very low frequency in B.1.1.529 lineage, suggesting selection of these mutations during the evolution of BA.2, Fig. 2f & h.

The spike region distinctly shows the highest concentration of mutations for Omicron lineages, yet observable differences in the mutation pattern do exist between BA.1 and BA.2. In comparison to previous VOCs, B.1.1.7 (Alpha) lineage exhibits an almost distinct spike profile having few overlaps with Omicron lineages at D614G, N501Y and P681H positions. As expected, B.1.617.2 and AY. * lineages share overlapping mutations with B.1.617.1. Notably, Delta and Omicron lineages also share overlaps in spike regions at positions T95I, G142D, T478K, P681R/H, respectively.

ORF3a majorly harboured low frequency mutations across all lineages, except S26L, found in high frequency in Delta and C25584T/64 T across Omicron lineages with T223I only in BA.2. The Omicron lineages have shown a high frequency mutation T9I in the E region. Like ORF3a, the M protein also showed presence of mutation at position 26767 (I82T) in Delta lineages. Likewise, in Omicron lineages, mutation Q19E and A63T of M region were commonly present in all the lineages with BA.2 having an additional synonymous mutation at C26858T/112 F. Delta lineages show multiple peaks in ORF6 and ORF7a whereas the other lineages show sparse presence of mutations in these regions. Moreover, ORF8 seems to be densely populated in Alpha. In N protein, mutations R203K and G204R are commonly shared between Alpha and Omicron lineages whereas Delta lineages carry only R203M.

Based on the relative size of each gene, Fig. 2b depicts the highest rate of mutations for each SARS-CoV-2 gene within Alpha, Delta, and Omicron. Omicron shows a completely different order of per gene mutation rates when compared to Alpha and Delta. Despite Spike having a high number of mutations in Omicron; ORF6, ORF8 and N protein show a higher mutation rate. ORF8 appears to have the highest mutation rate, whereas E protein appears to have the lowest in all three VOCs, with Alpha completely lacking mutation in E protein.

We further closely examined the variations between community samples and international travellers across different lineages (Table S2). In the community *vs* international samples of Delta plus (AY. *) lineage, we observed an overall higher number of non-clade defining mutations in community samples. These non-clade defining mutations were majorly unique between the two groups, except P2287S and P2046L. At the same time, two clade defining mutations, A222V and A1306S were present in international samples only (Fig. 2e). Contrarily, Omicron lineages showed a substantial overlap in the non-clade defining mutations between community and international samples pointing towards an early phase of transmission and evolution within the Indian community (Fig. 2f-h). Moreover, B.1.1.529 and BA.1 shows somewhat similar profile with mutations SL2083I, T5386G (A1707A), T13195C (V4310V), and C15240T (N591N) in the ORF1ab and del 69–70, 215EPE, L981F, N440K in Spike in both B.1.1.529 and BA.1 lineages of community and international travellers but absent from BA.2 lineage in our cohort, Fig. 2f-h.

Many other interesting mutation patterns are observed when comparing Omicron lineages. Mutation C2470T (A735A) shared between B.1.1.529 and BA.1 is absent from B.1.1.529 community samples, Fig. 2f. A non-clade defining mutation, R346I is invariably present in both community and international samples of BA.1 but completely absent from either B.1.1.529 or BA.2. Another important feature was the presence of four deletion mutations in spike protein and another del 3674–3676 within ORF1ab in the community samples of BA.1, which were absent in the international travellers. Mutation L3027F, L3201F, T2160I and E2196E (A20055G) of ORF1ab seen in only community samples of B.1.1.529 is interestingly seen in both community and international samples of BA.2 lineage, along with several other unique mutations. B.1.1.529 mutations, T376A, D405N, R408S eliminated in BA.1, are specifically selected in the BA.2 spike region. The observed variations in the spike mutations across VOCs as well as International and community samples need further assessment to unravel their functional consequences, impact on the transmission and pathogenicity of different VOCs.

### 3.3 Emergence of Signature spike mutations of different VOCs over time

We focused on the mutations present in the spike protein, due to the expansion observed in the Omicron variant as well as its emphasised role in infection and relevance to transmission and severity of disease. To identify mutation/s which might have evolved together in the different VOCs to synergistically provide advantage to the viral transmission, we performed LD analysis of all the spike mutations. We divided the samples into three groups based on time of sequencing and travel history as pre-Omicron Variants (n = 396) which included Alpha, Delta and Delta sub-lineages before the Omicron variant arrived in India; Community samples (n = 276) which included COVID-19 samples without any travel history between Dec2021-Jan 2022; and International travellers (n = 895) which included passengers who were COVID-19 positive on arrival at IGI Delhi airport from different countries, between Dec 2021-Jan 2022.

**Fig. 3:**
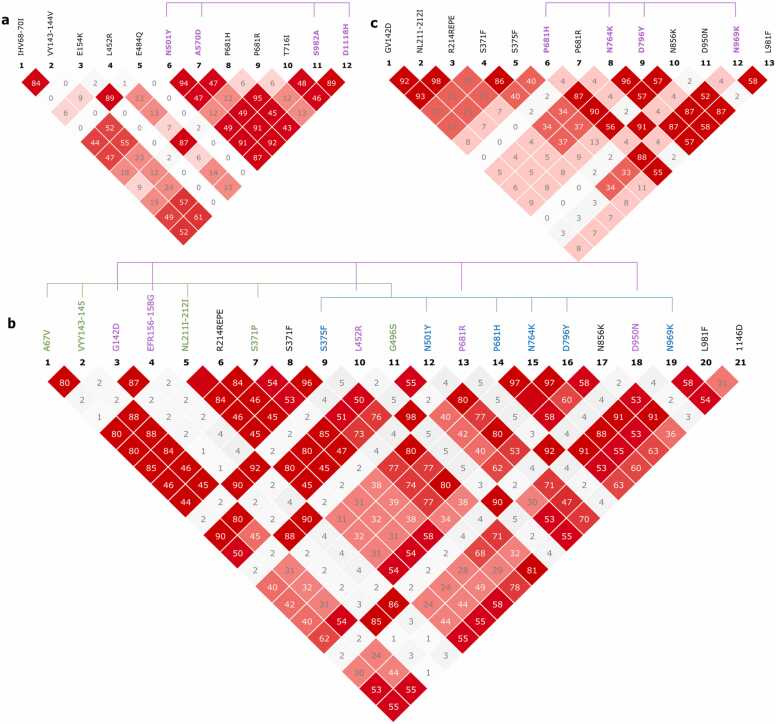
Linkage Disequilibrium analysis of Spike mutations in different variants over time in India. The LD plot represents spike mutations that are in strong LD with each other, with r2 > =0.8 and D′ > = 0.9. (a) In pre-omicron variants, (b) in community samples, and (c) in the international travellers’ groups. The colour of the plot represents r^2^ values as percentage.

**Fig. 4:**
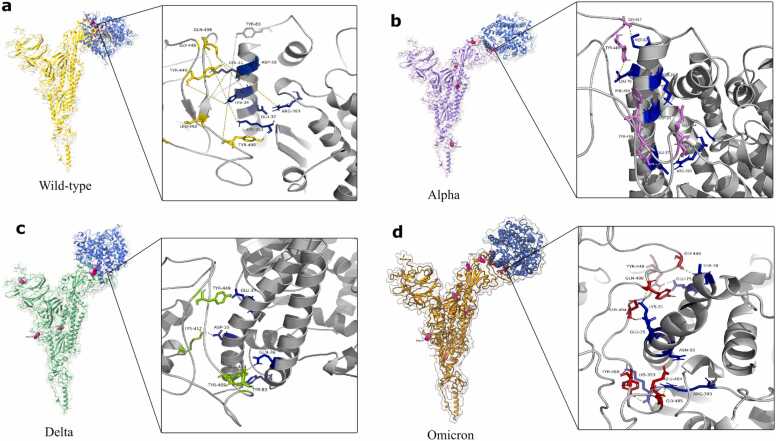
Docking representation of the Wild type and different variant mutation complexes. Illustrates the binding interfaces with key hydrogen interactions (a) wild-type complex, (b) Alpha variant complex, (c) Delta variant complex, and (d) Omicron variant complex.

LD analysis was performed separately for each of the three sub-groupings, considering all the spike mutations within each group with a MAF score > 0.005. We then filtered mutations which had D′ of > =0.9 and r^2^ > =0.8 (Table S3). Fig. 3 depicts the r^2^ values (in percent) for the mutations from each group that were filtered based on the previously indicated cut-off. In pre-Omicron variants, we found mutations [N501Y, A570D, D1118H and S982A] to be in strong LD with each other. All these are clade-defining mutations of Alpha and 97.5% of the samples shared all these mutations (Fig. 3a). In the community-transmission group, we found three separate sets of mutations in complete LD with each other. These were P681R, L452R, EFR:156–158 G, D950N, G142D which represents AY.* (Delta sub-lineage) mutations and 93.93% commonly shared all of these mutations; P681H, D796Y, N764K, N969K, N501Y, S375F which represents Omicron lineage (common mutations between B.1.1.529, BA.1 and BA.2) and G496S, S371P, A67V, GVYY142–145D, NL211–212I, 214EPE which represents mutations that belong uniquely to BA.1 lineage (Fig. 4b). In the International travellers’ group, we observed that majority of the filtered LD mutations [P681H, D796Y, N969K, N764K; GVYY142–145D, NL211–212I, 214EPE] overlapped with the Omicron community-transmissions group (Fig. 4c). Other mutation pairs in LD were P681R and D950N which represents the AY. * lineages; N856K, L981F and S371F, S375F which represent BA.1 and BA.2 lineages respectively (Fig. 4c). It seems that the spectrum of mutations in LD for different VOCs tended to expand subsequently during community transmission.

### 3.4 Uncovering structural features of the Spike mutations in different variants

The initial interaction of host-pathogen can be significantly altered due to mutations in the spike glycoprotein; thus, we investigated the effect of mutations on the binding efficiency of Spike-ACE2 receptor. We performed binding analysis of the wild-type and the mutants to evaluate their potential functional impacts. We selected five sets of spike mutations from the LD analysis, one for each of the three variants - Alpha, Delta and Omicron; and two for unique mutations of Omicron sub-lineages (BA.1 and BA.2), Table1. We used HADDOCK to perform protein-protein docking of ACE2 receptor with the wild-type spike protein (Set 0), mutations of Alpha (Set 1) [N501Y, A570D, S982A, D1118H], mutations of Delta (Set 2) [P681R, L452R, EFR:156–158 G, D950N, G142D], common mutations of Omicron (Set 3) [N501Y, P681H, D796Y, N764K, N969K, S375F]; to explore the structural mechanism behind the infectivity of the SARS-CoV-2 variants. We also performed docking with mutations of BA.1 lineage (Set 4) [G496S, S371P, A67V, GVYY142–145D, NL211–212I] and mutations of BA.2 lineage (Set 5) [T19I, V213G, T376A, R408S] considering both the unique and common mutations between the two lineages (Table S4).

Fig. 4 illustrates the visual differences in the docking patterns due to spike mutations in the wild-type and mutant complexes of Alpha, Delta and Omicron. The binding free energies range from − 14 (Delta) to − 10.7 (Alpha) and dissociation constant for all the mutations ranges from 0.05 nM to 15 nM. The binding free energy and dissociation constant of Delta (Set 2) (ΔG −14, K_D_ 0.05E-09), BA.2 (Set 5) (ΔG −11.9, K_D_ 1.90E-09) and Omicron (Set 3) (ΔG −12.2, K_D_ 1.20E-09) were found to be lower than the wild-type-ACE2 (Set 0) (ΔG −11.6, K_D_ 3.30E-09). However, the binding free energy and dissociation constant of BA.1 (Set 4) (ΔG −11.4, K_D_ 4.40E-09) and Alpha (Set 1) (ΔG −10.7, K_D_ 15.0E-09) were higher than the wild-type-ACE2 (Set 0), suggesting a weaker binding affinity between spike and ACE2 receptor for these VOCs.

**Fig. 5:**
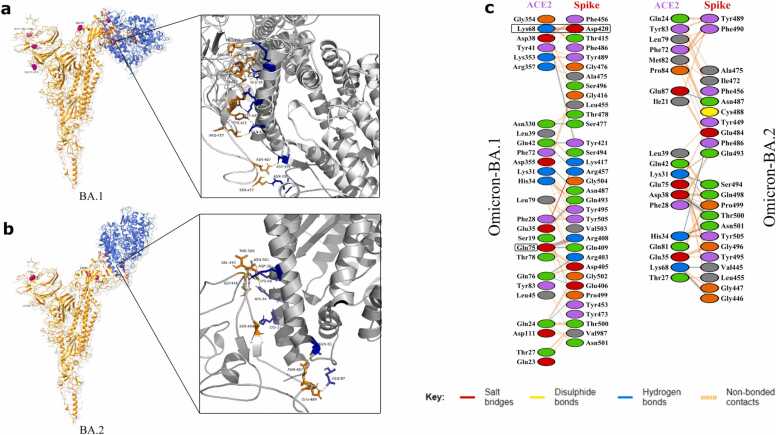
Docking model with binding interfaces and key hydrogen interactions from PDBsum. This includes for, (a) BA.1, and (b) BA.2 sub-lineages with ACE2 receptor. (c) 2D interaction representation of salt-bridges, hydrogen bonds and non-bonded interactions in BA.1 and BA.2 with ACE2 receptor.

To gain better insights into dissociation constant values (K_D_), interaction analysis was performed using PDBsum. The wild-type-ACE2 had one salt-bridge (between D30-K417) and seven hydrogen bond interactions (between K31-L492, H34-Y449, E37-Q498, Y83-Y489, K353-G446, R393-Q498), Fig. 4a. Alpha showed one salt-bridge (K26-E484) and five hydrogen bond interactions (D30-F490, E37-Y489, L79-Y449, M82-G447, R393-G485), Fig. 4b. Delta, although having strong binding free energy and low dissociation constant, had fewer interactions with one salt-bridge (between D30-K417), and only three hydrogen bond interactions (E37-Y449, Q76-E484, Y83-Y489), Fig. 4c. However, in the case of Omicron, Set 3 and Set 5 showed only hydrogen bond interactions, though both showed good binding free energy (K31-Y449, N33-E484, E35-S494, E75-Q498, T78-G446, K353-Y489, R393-G485), Fig. 4d and Fig. 5b. Finally, Set 4 (BA.1) showed the highest number of interactions at three salt-bridges and 13 hydrogen bond interactions Figs. 5a and 5c.

## 4 Discussion

In the current study, we present a comprehensive comparative genomic landscape of the major SARS-CoV-2 VOCs prevalent in India from Dec 2020-Jan 2022, Alpha, Delta and Omicron, as well as explore the differences in mutation profile between international travellers and community samples. On 18th December 2020, more than a year into the pandemic, the first “variant of concern” was identified by WHO, based on the epidemiological, immunological and pathogenic properties of the circulating variant and now in the third year of the pandemic, five different prominent VOCs (Alpha, Beta, Gamma, Delta and Omicron) and multiple variants of interest (VOI) have been reported (Tracking SARS-CoV-2 variants [Internet]). Though all of them were not equally challenging globally as well as for India, for example, ~4 million cases of Delta have been reported worldwide whereas only ~1 million are reported for Alpha variant, although it has existed for a longer time than Delta (Outbreak.info). Similar country-wide trend is reflected in our cohort data; where the Alpha variant was swiftly supplanted by the Delta, which persisted for a long period, until the entry and emergence of Omicron in the subcontinent, Fig. 1a. However, since our data was part of active surveillance of international travellers along with community transmission, it reflected the trend for international travellers too. Hence, our cohort data showed a higher prevalence of B.1.1.529 followed by BA.1 then BA.2, although country-wide trends and studies showed a quick dominance of BA.2 over B.1.1.529 and BA.1 from Dec 2021 to Jan 2022 (Fig. 1b) (Dhawan et al., 2022 Mar). It is interesting to note that both Alpha and Omicron were first detected in India in the month of December (albeit different years) and quickly faded off which is reminiscent of a pattern of transmission. The phylogenetic analysis also showed diversification of the Omicron variant from the same parental branch as that of Alpha Fig. 1c. Nevertheless, the huge difference in the number of mutations especially in the spike region between the two raises concern for behavioural similarity. This led us to deeply investigate the overall mutational landscape as well as delve deeper into the role of spike mutations between the different VOCs.

Mutations at six positions were identified to be prevalent across all the VOCs: one in the 5′ UTR: C241T; two in ORF1ab: C3037T and P314L; two in S: D614G, P681H/R; and one in N: R203K/M, indicative of their positive selection in emerging lineages Fig. 2c-h. Although clade-defining mutations are widespread in both International and community samples, the A222V mutation was found in < 10% of Delta plus community samples (Table S2). Following that, discrepancies in non-clade defining mutations were discovered, particularly between International and community samples of the Delta plus lineage, Fig. 2e. Mutations in ORF1ab: P271S, K1763N, S2048F, and K2705R were found only in community samples, whereas mutations in ORF1ab: K261N, A2529V and L829I; and ORF7a: P45L were found in international samples. Although not much is known about these mutations, ORF1ab: K261N and ORF7a: P45L is reported to be prevalent among 90% of AY.122 lineages from Russia(Klink et al., 2022). These findings suggest that the virus is continuously expanding in the evolutionary space. Moreover, the periods of low COVID-19 cases, as witnessed during the Delta plus lineage time, might be a part of a silent spreading event wherein the virus may be optimising different combinations of mutations which when complimented with perfect micro-environment provided by the immuno-compromised host and favourable replication conditions, lead to the emergence of new VOCs (Moghadas et al., 2020).

The Omicron variant is known to contain several key spike mutations such as K417N, E484A, N440K, N501Y and P681H, which are also observed in other SARS-CoV-2 VOCs, albeit the presence of many novel mutations alongside complicates the functional relevance of these mutations to disease outcome. Initial studies on Omicron revealed its highly infectious and transmissible nature along with immune evasion properties ((Potential Rapid Increase of Omicron Variant Infections in the United States | CDC [Internet],) Araf et al., 2022 May), yet the milder clinical presentation raised curiosity to decipher whether the combined effect of the spike mutations is responsible for ameliorating the disease manifestation. Several Omicron spike mutations reportedly enhanced the binding to ACE2, whereas others were found to have a neutral effect or reduced binding (Starr et al., 2020). Possibility exists that combination of spike mutations can alter ACE2's affinity for binding (Yan et al., 2020). A recent study suggested that RBD combinations of Omicron existed which varied with geographical patterns (Ou et al., 2022). The structural differences of mutant spike and ACE2 binding complex were investigated using this background important knowledge. Linkage Disequilibrium analysis utilising spike mutations present in VOCs from different time points and between community and international travellers’ samples identified a combination of mutations that were inherited together in different variants Fig. 3. Binding interaction of the ACE2 receptor with these combinations of spike mutations provided evidence which corroborated with the transmissibility and infectivity observed for Alpha, Delta and Omicron Table 1.

**Table 1:** Docking result for selected mutation groups arranged in ascending order of dissociation constant (K_D_).

**Protein-Protein Complex**	**Mutations**	**Lineage**	**Binding free energy ΔG (kcal mol-1**	**Dissociation constant Kd (nM) at 25.0 ℃**	**Interacting residues**
**Set 0**	Wild type	Ref	-11.6	3.30E-09	105
**Set 1**	N501Y, A570D, S982A, D1118H	Alpha (B.1.1.7)	-10.7	15.0E-09	81
**Set 2**	P681R, L452R, EFR156–158 G, D950N, G142D	Delta (B.1.617.2, AY. *)	-14	0.050E-09	92
**Set 3**	N501Y, P681H, D796Y, N764K, N969K, S375F	Omicron (B.1.1.529, BA.1 & BA.2)	-12.2	1.20E-09	80
**Set 4**	G496S, S371P, A67V, GVYY142–145D, NL211–212I	BA.1	-11.4	4.40E-09	133
**Set 5**	T19I, V213G, T376A, R408S	BA.2	-11.9	1.90E-09	83

Alpha revealed four mutations: N501Y, A570D, S982A, and D1118H to have high LD. It demonstrated very high binding free energy (thus a lower binding affinity) with one salt-bridge between K26-E484 residues, and also share a hydrophobic bond with K26-I472, implying a trade-off of the ionic interaction, resulting in a higher binding energy than any other variants Fig. 4b. Overall, only one of four selected mutations fell within the receptor binding domain (RBD); and amino acid changes mostly resulted in hydrophobic residues that reduce physical interactions and further avoid contact with the solvent (Hou et al., 2018), thus affecting the RBD binding interface.

Delta showed four substitution mutations and one deletion; EFR: 156–158 G, P681R, L452R, G142D, D950N occur in the N-terminal domain (NTD), RBD, cleavage site, and Heptad repeat 1 (HR1), respectively whereas Omicron mutations: S375F, N501Y, P681H, N764K, D796Y, N969K, occur in RBD, cleavage site, fusion peptide, and HR1 (heptad repeat), respectively Fig. 4c-d. The binding energy of Delta was the lowest with one salt-bridge interaction between D30-K417, and along with several hydrogen bonds interactions whereas Omicron also had binding energy lower than the wild-type but did not show any salt bridge interaction. The mutations in Delta and Omicron mostly occupied the functional domain (NTD, RBD), with positively charged amino acids, fulfilling the alkaline requirement by the variant for enhanced binding with the ACE2 receptor (Nie et al., 2021; Kumar et al., 2022 Apr), thus supporting the high transmission rate observed for Delta and Omicron.

BA.1 showed three mutations and two deletions in LD: A67V, S371P, G496S, NL211–212I, GVYY142–145D falling in NTD and RBD. BA.2 showed four mutations, T19I, V213G, T376A, R408S, also in NTD and RBD. The binding energy of BA.1 is higher than wild type, while BA.2 is lower, indicating BA.2 has a better binding with the ACE2 receptor than BA.1 Fig. 5. In BA.1, interactions have three salt-bridges E35-K417, K68-D420, and E75-R408 and hydrophobic interactions at the same residues, E35-Y453, K68-Y421, and E75-D405, whereas BA.2 does not show any salt-bridge interaction. Interestingly, all the mutations in BA.1 and BA.2 are occurring in the functional domain, yet higher binding free energy in BA.1 than wild type may result from the mutations’ trade-off with two types of interactions occurring in the same residue. Moreover, the presence of many deletions in BA.1, mostly in the NTD region, might have led to structural modifications, which are significantly distinct from those seen in previous variants. These deletions also hold potential to considerably alter the antigenic surface of the NTD uniquely, explaining the loss of binding and neutralisation by NTD-1 antibodies (Zhang et al., 2022).

The reduced binding energy observed for Delta, Omicron and BA.2 variants compared to the wild-type implied the presence of strong binding between spike-ACE2, whereas Alpha and BA.1 displayed higher binding energy than the wild-type suggesting reduced binding affinity for ACE2. This could predict the sudden supplant of Delta over Alpha and BA.2 over BA.1 in India. Taking together, the findings of mutational positions, altered amino acid composition, downstream docking and interaction analysis, we hypothesise the combined functional impact of these mutations correlating with the dissemination of each of the VOCs.

## 5 Conclusion

Our study highlights the effectiveness of Genomic surveillance that helped to track and decode the mutation landscape of the VOCs: Alpha, Delta and Omicron, upsurge in India during different time points. We have elucidated the spatio-temporal behavioural transmission patterns of the variants based on the selective advantage incurred by the spike mutations for a likely initial binding event to the host cell, leading to infectivity and transmissibility. The findings are relevant for the future with the possibility of a new VOC/VOI.

## Ethics approval and consent to participate

The studies involving human participants were reviewed and approved by CSIR-IGIB’s Human Ethics Committee Clearance (Ref No: CSIR-IGIB/IHEC/2020–21/01). The patients/participants provided their written informed consent prior to participation in this study.

## Funding support

This research was funded by the 10.13039/100000865Bill and Melinda Gates Foundation (BMGF), Grant number [INV-033578], Foundation for Innovative New Diagnostics (FIND), project code [GAP-0249], and AIDS Healthcare Foundation (AHF), project code [CLP-0043].

## Data Availability

The clinical dataset collected and analysed as a part of this study is attached as.

## CRediT authorship contribution statement

**Priyanka Mehta**: Methodology, Formal analysis, Data curation, Writing − original draft, Visualisation. **Varsha Ravi**: Methodology, Formal analysis, Data curation, Writing − original draft, Visualisation. **Priti Devi**: Investigation. **Rajeet Maurya**: Formal analysis, Data curation. **Shaista Parveen**: Investigation. **Pallavi Mishra**: Formal analysis, Data curation. **Aanchal Yadav**: Investigation. **Aparna Swaninathan**: Investigation. **Sheeba Saifi**: Investigation. **Kriti Khare**: Investigation. **Partha Chattopadhyay**: Investigation. **Monika Yadav**: Investigation, Data curation. **Nar Singh Chauhan**: Investigation, Formal analysis, Data curation, Supervision. **Bansidhar Tarai**: Data curation. **Sandeep Bhudiraja**: Supervision. **Uzma Shamim**: Conceptualisation, Methodology, Formal analysis, Writing − original draft, Project administration. **Rajesh Pandey**: Conceptualisation, Methodology, Writing − original draft, Project administration, Funding acquisition.

## Conflict of Interest

Authors wish to declare no conflict of interest and funders did not have a role in planning and execution of the study.
